# Histopathology of *Pneumocystis carinii* pneumonia in immunocompetent laboratory rats

**DOI:** 10.3892/etm.2014.1732

**Published:** 2014-05-26

**Authors:** HYUN-SOO KIM, SUNG-IM DO, YOUN WHA KIM

**Affiliations:** 1 Department of Pathology, Samsung Medical Center, Sungkyunkwan University School of Medicine, Seoul 135-710, Republic of Korea; 2 Republic of Korea Air Force Aerospace Medical Center, Chungcheongbuk-do 363-849, Republic of Korea; 3 Department of Pathology, Kangbuk Samsung Hospital, Sungkyunkwan University School of Medicine, Seoul 110-746, Republic of Korea; 4 Department of Pathology, School of Medicine, Kyung Hee University, Seoul 130-701, Republic of Korea

**Keywords:** *Pneumocystis carinii*, rat, lung, rat respiratory virus, interstitial pneumonia

## Abstract

The occurrence of idiopathic pulmonary lesions in laboratory rats, characterized by lymphohistiocytic interstitial pneumonia with dense perivascular lymphoid cuffs, has been reported over the past decade. Although the term rat respiratory virus (RRV) was adopted to confer a putative viral etiology to the idiopathic pulmonary lesions, the etiology of this disease remains to be elucidated. Recently, inflammatory lesions have been observed in the lungs of immunocompetent laboratory rats similar to those previously described. Based on the latest evidence indicating that *Pneumocystis carinii (P. carinii)*, and not putative RRV, causes infectious interstitial pneumonia in laboratory rats, the present study investigated whether the pulmonary lesions observed were caused by *P. carinii* infection. Male Sprague-Dawley rats, free of known pathogens, were introduced into a rat colony positive for RRV-type lesions. Routine histopathological examinations were performed on the rat lung tissues following exposure. The presence of *Pneumocystis* organisms was confirmed using Grocott’s methenamine silver (GMS) staining. At week 3 following introduction, a few small lymphoid aggregates were located adjacent to the edematous vascular sheath. By week 5, foci of dense perivascular lymphoid cuffing were observed. Multifocal lymphohistiocytic interstitial pneumonia and prominent lymphoid perivascular cuffs were observed between week 7 and 10. GMS staining confirmed the presence of *Pneumocystis* cysts. Thus, the results of the present study demonstrated that *P. carinii* caused lymphohistiocytic interstitial pneumonia in a group of laboratory rats. The observations strongly support the conclusion that *P. carinii* infection in immunocompetent laboratory rats causes the lung lesions that were previously attributed to RRV.

## Introduction

Health monitoring of laboratory animals used in biomedical research is necessary due to the consequences of unwanted infections. Animals that appear normal and healthy may be unsuitable as research subjects due to unobservable, but significant local or systemic effects of viruses, bacteria, fungi and parasites with which they may be infected. There has been a steady increase in the awareness of the varied and generally unwanted effects of natural pathogens in laboratory animals, and there have been even greater efforts to exclude pathogens from these animals (1). Valid experimental data can only be generated and interpreted when laboratory animals are free of pathogens that may alter the host physiology.

In the late 1990s, a series of studies emerged describing the occurrence of idiopathic pulmonary lesions, characterized by lymphohistiocytic interstitial pneumonia with prominent lymphoid perivascular cuffs, in laboratory rats (2,3). The lymphohistiocytic nature and eventual resolution of the lesions indicated an infectious etiology. Although attempts to identify the etiology of this disease have been unsuccessful, a previous study detected a limited cytopathic effect in cell lines that had been incubated with lung tissue homogenates from diseased rats, and the pulmonary lesions were reproduced in rats inoculated with the cultured cells (4). In conjunction with the perivascular and interstitial distribution of the lesions and the lack of an identifiable etiologic agent, these observations led to the tentative conclusion that the lesions were caused by a novel rat virus. The term rat respiratory virus (RRV) was adopted to confer a putative viral etiology to the idiopathic pulmonary lesions and has been used over the past decade (5–8). In a study of rats from research institutions in North America, ~6% of the rats evaluated exhibited histological evidence of idiopathic pulmonary lesions consistent with RRV infection (RRV-type lesions) (9).

However, previous studies have provided convincing evidence that *Pneumocystis carinii (P. carinii)*, and not putative RRV, causes infectious interstitial pneumonia in laboratory rats (7,8). Livingston *et al* (7) demonstrated that *Pneumocystis* DNA was present in the lungs of several immunocompetent rats with RRV-type lesions. In addition, Henderson *et al* (8) confirmed a causative association between *P. carinii* infections and the time course and severity of interstitial pneumonia in immunocompetent laboratory rats. Consequently, these authors have proposed this prevalent, once idiopathic disease, to be called *P. carinii* pneumonia (PCP). *P. carinii* is a single-celled fungal respiratory pathogen of mammals that is transmitted via an airborne route (10,11). Although *P. carinii* is known to cause a severe and often lethal pneumonia in immunocompromised hosts (12–14), several studies have found PCP in immunocompetent hosts, including rats and mice naturally or experimentally infected with *Pneumocystis* spp. (5,15–19).

Recently, inflammatory lesions have been observed in the lungs of immunocompetent laboratory rats, very similar to those previously described (6–8). Since *P. carinii* has been detected in a wide variety of commercial rat colonies (20,21), assessing the presence of *Pneumocystis* infections in rats is necessary prior to their experimental use. Based on the latest evidence indicating that *P. carinii* causes the infectious interstitial pneumonia that was previously attributed to RRV in laboratory rats (7,8), the present study investigated whether the lesions observed were caused by *P. carinii* infection. To evaluate the transmissibility and time course of the lesions, naive rats were introduced into an endemically infected colony and the pulmonary histopathology was assessed over a 14-week period.

## Materials and methods

### Animals

Male Sprague-Dawley rats weighing 250–260 g were obtained from Samtako Bio Korea, Inc. (Osan, Korea). Routine monitoring of the sentinels found no evidence of antibodies to the Theiler’s meningoencephalitis virus group, hantavirus, Kilham rat virus, pneumonia virus of mice, rat coronavirus, rat minute virus, rat parvovirus, sialodacryoadenitis virus, Sendai virus, Toolan’s H-1 virus, reovirus, lymphocytic choriomeningitis virus, rat adenovirus, cilia-associated respiratory bacillus, *Mycoplasma pulmonis* and *Clostridium piliforme*. In addition, routine cultures grown on appropriate media were negative for β-hemolytic *Streptococcus*, *Bordetella bronchiseptica*, *Corynebacterium kutscheri*, *Escherichia coli* (as determined by serotyping), *Helicobacter* spp. [as determined by polymerase chain reaction (PCR)], *Klebsiella oxytoca*, *K. pneumoniae*, *Pasteurella pneumotropica* and other *Pasteurella* spp., *Pseudomonas aeruginosa*, *Salmonella* spp., *Staphylococcus aureus*, *Streptobacillus moniliformis* and *S. pneumoniae*, amongst other pathogenic bacteria. Examination of the pelage and gastrointestinal tract found none of the following parasites: *Aspicularis* spp., *Eimeria* spp., *Entamoeba muris*, *Giardia muris*, *Hymenolepis* spp., *Klossiella muris*, *Liponyssus* spp., *Myobia* spp., *Myocoptes* spp., *Notoedres* spp., *Polyplax* spp., *Psorergates* spp., *Radifordia* spp., *Rodentolepis nana*, *Spironucleus* spp., *Syphacia* spp., trichomonads and *Trichosomoides crassicauda*.

### Colony

Naive rats were introduced into a rat colony positive for RRV-type lesions, as previously described (6). The colony was negative, prior to and following the duration of the study, for all the pathogens listed previously for the naive rats. The presence of RRV-type lesions was determined via histopathological evaluation of the lung tissue for characteristic inflammatory lesions, according to established diagnostic criteria (6). Board-certified pathologists confirmed the presence of RRV-type lesions in the colony animals during multiple evaluations. The rats were housed 3–4 per cage in wire-top solid-bottom cages located on the bottom racks at various locations throughout a room maintained at 22±0.5°C with a 12 h light-dark cycle. Bedding in the rat cages (Samtako Bio Korea, Inc.) consisted of a 50:50 mix of clean and soiled hardwood bedding from the colony animals. The soiled bedding of the positive colony was collected at routine bedding changes, whereas the bedding of the negative control animals were replaced with fresh materials. All the rats had free access to standard rat chow (Samtako Bio Korea, Inc.) and water throughout the study.

### Histopathological examination

To investigate the time course of the pulmonary lesions, rats were randomly divided into seven group consisting of 9–12 rats per group. At each time point [0 (rats were purchased and transferred to the necropsy laboratory directly after receipt] and at week 1, 3, 5, 7, 10 and 14 following introduction into the room), each group of rats was anesthetized with 50 mg/kg sodium pentobarbital (Hanlim Pharm. Co., Ltd., Seoul, Republic of Korea) intraperitoneally, and then laparotomized via a midline incision. Following laparotomy, the bilateral lungs were removed from the thoracic cavity and immediately preserved in 10% neutral-buffered formalin solution (Sigma-Aldrich, Co., LLC, St. Louis, MO, USA). After 48 h of formalin fixation, the lung tissue samples were embedded in paraffin and processed for routine hematoxylin and eosin (HE) staining. Grocott’s methenamine silver (GMS) staining was performed to confirm the presence of *Pneumocystis* organisms. Board-certified pathologists examined the HE- and GMS-stained lung sections using a light microscope (BX51; Olympus Optical Co., Ltd., Tokyo, Japan) and morphological observations were recorded. All the procedures were conducted in accordance with the Guide for Care and Use of Laboratory Animals published by the National Institutes of Health and the ethical guidelines of the International Association for the Study of Pain. The institutional animal ethics committee of the Republic of Korea Air Force Aerospace Medical Center (Cheowgwon-gun, Chungcheongbuk-do, Republic of Korea) approved all experimental procedures involving animals.

## Results

### Clinical features

At week 7, the rats exhibited variable retardation in weight gain. By week 10, two rats showed moderate dyspnea and severe weight loss. None of the rats exhibited severe respiratory distress or marked changes in behavior during the experiments.

### Gross findings

No gross pulmonary lesions were observed in the rats between weeks 0 and 5. Gross pulmonary lesions were first identified at week 7 and by week 10, all the rats had gross pulmonary lesions. The lungs of the rats were slightly enlarged and moderately consolidated with a mottled appearance, or interspersed with single or multiple gray or white foci of increased firmness. Grossly visible necrosis, hemorrhage or pleural thickening was absent.

### Microscopic findings

**Figure 1:**
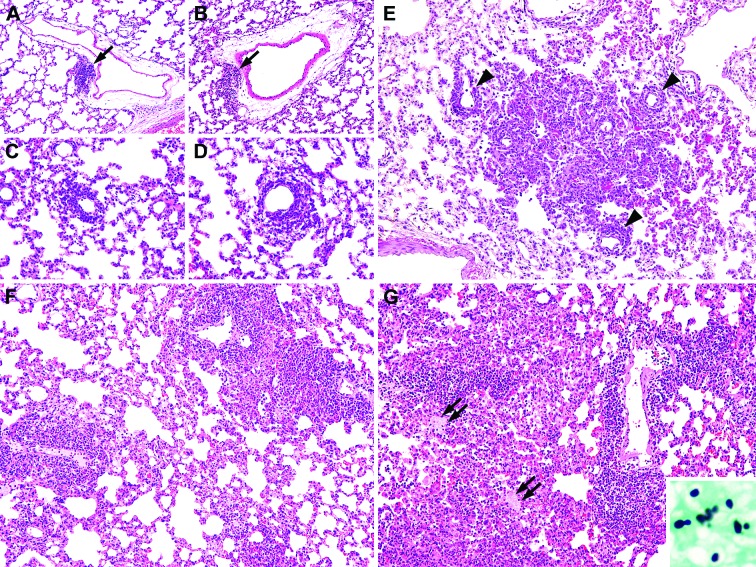
Histopathology of PCP in immunocompetent laboratory rats. (A and B) At week 3 following introduction, the earliest change was the appearance of edema with a few small lymphoid aggregates located adjacent to the edematous vascular sheath (single arrows). (C and D) At week 5, dense lymphoid cuffs surrounded the blood vessels and the adjacent alveolar septa were minimally infiltrated by lymphocytes. (E) At week 7, areas of lymphohistiocytic interstitial pneumonia showing alveoli infiltrated by lymphocytes and macrophages and thickened alveolar septa were observed. Dense perivascular lymphoid cuffs were more frequently identified (arrowheads). (F) At week 10, thick bands of lymphocytes and macrophages encircled the blood vessels adjacent to the areas of alveolar septal thickening and leukocytic infiltrates. (G) Lymphohistiocytic interstitial inflammation led to septal thickening with extensive alveolar leukocytic infiltrates and the formation of dense cuffs and aggregates of lymphocytes around the blood vessels. Foamy eosinophilic exudates were also observed (double arrows) and a number of GMS-positive *Pneumocystis* cysts (inset), characteristic of PCP, were identified in the same areas as the interstitial pneumonia. (A–G) Hematoxylin and eosin staining and (G; inset) GMS staining. (A and B) magnification, ×40; (C–G) magnification, ×100; and (G; inset) magnification, ×400. PCP, *Pneumocystis carinii* pneumonia; GMS, Grocott’s methenamine silver.

No microscopic changes were observed in the lungs of the rats during weeks 0 and 1. However, by week 3, pulmonary blood vessels exhibited an edematous vascular sheath with a few small lymphoid aggregates surrounding the vessels (Fig. 1A and B). The adjacent alveolar spaces and septa exhibited no pathological abnormality. By week 5, dense lymphoid cuffs surrounded the blood vessels (Fig. 1C and D) and the adjacent alveolar septa were minimally infiltrated with lymphocytes and macrophages. By week 7, the lymphoid perivascular cuffs were more frequently identified. In addition, areas of nonsuppurative lymphohistiocytic interstitial pneumonia showing alveoli infiltrated by lymphocytes and macrophages and thickened alveolar septa were observed (Fig. 1E). Perivascular hemorrhage was also observed at this time point. By week 10, a substantial increase in perivascular lymphocytes and macrophages formed thick leukocytic cuffs encircling the blood vessels adjacent to the areas of alveolar septal thickening and leukocytic infiltrates (Fig. 1F). Compensatory alveolar emphysema was also identified. The infiltrating cells were primarily lymphocytes and macrophages, with a small number of eosinophils and plasma cells. The lesions consisted of marked lymphohistiocytic interstitial inflammation, extensive alveolar leukocytic infiltrates with septal thickening and the formation of dense lymphoid cuffs and aggregates around the small blood vessels. In a few foci, foamy eosinophilic exudates characteristic of PCP were also identified (Fig. 1G). GMS staining showed a number of typical ovoid, thick-walled *Pneumocystis* cysts (Fig. 1G; inset), but no airway changes were identified. By week 14, the lesions were resolved in about half of the rats. No pathological abnormality was observed in the control animals.

## Discussion

Health surveillance of laboratory animals is conducted since adventitious infection is a realistic possibility that can have a significant, negative impact on animal research (9). Although infectious interstitial pneumonia, a prevalent and transient interstitial pneumonia of immunocompetent laboratory rats, had been attributed to a putative virus referred to as RRV, the etiology of infectious interstitial pneumonia has not been established (4,22). In the present study, naive rats housed on 50% soiled bedding from the cages of colony rats with RRV-type lesions were shown to develop lesions identical to those of the colony animals. In addition, *P. carinii* was found to be an etiologic agent of infectious interstitial pneumonia in a group of immunocompetent laboratory rats. The observations support previous data that demonstrated the transmissibility of the condition. Widespread presence of *P. carinii* has been documented in the lungs of clinically-healthy, commercially-produced immunocompetent rats (5,23). The infectivity of *P. carinii* in immunocompetent laboratory animals has also been supported by previous studies (7,8,15,16,19,24). Henderson *et al* (8) demonstrated that *P. carinii* infection was transmitted to immunocompetent rats by bedding transfer and direct contact with contagious animals. In addition, Gigliotti *et al* (16) demonstrated that brief cohousing with *P. carinii*-infected mice resulted in infected immunocompetent mice. There was active replication of the organisms in the immunocompetent host such that the organism was transmitted to other *Pneumocystis*-free immunocompetent mice, again with active replication. Similarly, Chabé *et al* (19) found that healthy host-to-healthy host transmission of *Pneumocystis* organisms can occur, and that *Pneumocystis* organisms are able to replicate in the lungs of immunocompetent hosts, indicating that these hosts are a reservoir for *P. carinii*. The observations of the present study are consistent with these studies.

In addition, the results of the present study provide a histopathological description of the PCP time course in immunocompetent rats. Consistent with previous results (6–8), characteristic lesions of PCP were observed, consisting of multifocal lymphohistiocytic interstitial pneumonia and dense perivascular cuffs of lymphocytes and macrophages around the blood vessels. Foamy eosinophilic exudates containing GMS-positive *Pneumocystis* cysts were also detected. Gross lesions appeared during week 7, while the microscopic lesions necessary for diagnosis appeared slightly earlier at week 5. The time course of the observations indicated that the lesions were mild at week 5, severe lesions appeared between week 7 and 10 and resolution occurred by week 14 following exposure. This time course is similar to that observed in a previous study by Albers *et al* (6), but not consistent with that of a recent study by Livingston *et al* (7) that found that the lesions in rats were first observed at week 3, most severe at week 5 and were resolved by week 7. The reason for this difference in lesion onset, progression and resolution is unknown, but may reflect several factors, including the timing of infection, strain of *Pneumocystis*, strain of rat and other environmental factors.

It is well-known that immunocompetent rats can be subclinically infected with *Pneumocystis* spp., but the development of PCP was considered to occur only in immunodeficient rats due to genetic factors (for example, nude rats) or artificial interventions (for example, immunosuppressive doses of glucocorticoids) (5,23,25). Thus, immunocompetent rat colonies have not been routinely tested for *Pneumocystis* infections. Now that an etiology for RRV-type lesions has been identified, *Pneumocystis*-specific diagnostic tests, including histopathological examination for the identification of pneumonia and other organisms and PCR assays for the detection of *Pneumocystis* DNA, can be used to detect infected rats and monitor colonies for *Pneumocystis* infections. Previously, histopathology was the only diagnostic assay available to detect rats with idiopathic pneumonia, now known as PCP (6). However, high-throughput immunoassays to detect serum antibodies against *Pneumocystis* and quantitative PCR to quantify *Pneumocystis* DNA expression may also be used in the testing of rat colonies for this pathogen.

Although the present study documented the role of *P. carinii* as the causative agent of significant lung pathology in immunocompetent rats, it is unknown whether other *Pneumocystis* spp. are capable of causing a similar disease. *P. carinii* and *P. wakefieldiae* are the two *Pneumocystis* spp. that have been documented to infect rats used in biomedical research, however, at least three other provisional *Pneumocystis* spp. have been identified in wild rats (25,26). *P. carinii* can occur as a monoinfection or coinfection with *P. wakefieldiae* (5,26). The possibility that other *Pneumocystis* spp. cause pneumonia in immunocompetent rats cannot be eliminated, thus, excluding all *Pneumocystis* spp. from rat colonies in which *Pneumocystis* pneumonia is unwanted is recommended. Further investigation is necessary to address the pathogenic potential of *P. wakefieldiae* in immunocompetent rats.

It is well established that *P. jirovecii* infections cause severe pneumonia in immunocompromised humans and are a leading cause of mortality in patients with acquired immunodeficiency syndrome. However, evidence is starting to be amassed associating subclinical *P. jirovecii* infections in immunocompetent humans with several diseases in infants and adults, including sudden infant death syndrome, chronic obstructive pulmonary disease, asthma, bronchiolitis and other lung conditions (27). The marked, predictable lung pathology in immunocompetent laboratory rats that are naturally infected with *P. carinii* may provide a possible animal model for *P. jirovecii* infection in immunocompetent humans.

Limitations of the data presented in the current study should be acknowledged, including the presence of sampling error. The data are merely compilations of results from the sample stream passing through the laboratory and were not selected as representative samples from entire populations. In addition, since the observations are derived almost exclusively from the testing of laboratory rats, typically of a specific pathogen-free health status, the data cannot be construed to indicate that rodents reared as pets or to feed raptors or reptiles are of a similar health status as specific pathogen-free rats. Furthermore, as the sources of *P. carinii* for the experiments were infected rat lungs and not pure cultures, it cannot be claimed categorically that no other agent is involved in the pathogenesis of interstitial pneumonia. For instance, a virus transmitted with *P. carinii* may contribute to the disease by facilitating *Pneumocystis* colonization of the lower respiratory tract in immunocompetent hosts. However, the participation of an infectious agent other than *P. carinii* in the pathogenesis of interstitial pneumonia is highly improbable for the following reasons. Firstly, routine surveillance of the colony used in the experiments did not detect any known pathogens. Secondly, novel pathogens are infrequently found in long-used and intensively characterized laboratory animal species, such as the rat.

In conclusion, the results of the present study demonstrate that *P. carinii*, and not a virus, is the causative agent of lymphohistiocytic interstitial pneumonia in a group of laboratory rats. The observations strongly support the conclusion that *P. carinii* infection in immunocompetent laboratory rats causes the lung lesions that were previously attributed to RRV.
